# The association between polypharmacy and late life deficits in cognitive, physical and emotional capability: a cohort study

**DOI:** 10.1007/s11096-018-0761-2

**Published:** 2018-11-29

**Authors:** Mina Khezrian, Christopher J. McNeil, Phyo K. Myint, Alison D. Murray

**Affiliations:** 1 Aberdeen Biomedical Imaging Centre, Institute of Medical Sciences, University of Aberdeen, Foresterhill, Aberdeen, AB25 2ZD UK; 2 Ageing Clinical and Experimental Research, Institute of Applied Health Sciences, University of Aberdeen, Foresterhill, Aberdeen, AB25 2ZD UK

**Keywords:** Cognitive impairment, Emotional impairment, Observational cohort study, Older adults, Physical impairment, Polypharmacy, United Kingdom

## Abstract

*Background* Polypharmacy is a growing health concern for older adults and is associated with poorer clinical outcome. *Objective* This study aim is to investigate the association between polypharmacy and impairment in cognitive, physical and emotional capability controlling for the confounding effect of co-morbidities. *Setting* The Aberdeen 1936 Birth Cohort from 1999 to 2004. *Method* Recruited were 498 dementia free participants around 64 years old and recruited into wave one. Linear regression and structural equation models were used. Models were adjusted for the effect of age, gender, childhood IQ, education and Body Mass Index. A triad of impairment was defined as a composite measure of impairment in cognitive, physical and emotional function. *Main outcome measure* The relationships between polypharmacy, co-morbidity and triad of impairment. *Results* The prevalence of polypharmacy was 12.3% in this relatively healthy sample. Polypharmacy was significantly associated with increased impairment in cognitive, physical and emotional ability (β = 3.6, *p* = 0.003) after controlling for the effect of comorbidities and other confounding variables. As expected, higher childhood IQ and educational achievement had protective effects against impairment while higher comorbidity score and Body Mass Index were associated with increased impairment in this population. *Conclusions* The independent association of polypharmacy and reduced cognitive, physical and emotional capability makes this a promising target for predicting and potentially reducing the risk of impairment and associated healthcare costs in older adults. Longitudinal studies are required to investigate the underlying mechanisms for the observed relationships further.

**Electronic supplementary material:**

The online version of this article (10.1007/s11096-018-0761-2) contains supplementary material, which is available to authorized users.

## Impacts on practice

- Considering number of medications could improve our understanding of functional health status in older adults.
- Interventions to reduce inappropriate polypharmacy might improve cognitive, physical and emotional capability and outcomes in the elderly.

## Introduction

Aging is associated with the accumulation of multiple chronic diseases and increased risk of varying degrees of physical, cognitive and emotional impairment [1]. Prescribing medicines is still the most common form of medical intervention to manage chronic diseases and many older people will take several prescription medications. Polypharmacy, which is usually defined as taking five or more medications [2], is a common consequence of the increasing co-morbidity burden seen in older age. Polypharmacy could cause increased side effects, such as falls and harmful medicine interactions in older people [3]. Polypharmacy may also increase the risk of cognitive and physical impairment and frailty, in elderly people independently of other risk factors [4–6].

Decline in cognitive and emotional function frequently co-exists with physical dysfunction in late life [7, 8]. Common pathological pathways such as macroinfarcts and nigral neuronal loss in the brain, hormonal changes and inflammation may explain the co-occurrence of mental and physical impairment in older adults [9, 10]. Investigating the shared impairment in cognitive, physical and emotional function, or the “triad of impairment” (TOI) [11–13], is an approach to quantifying such decline. This approach could make these complex inter-relationships between impairment in multiple domains more accessible for clinicians to quantify and address in treatment and prevention strategies.

### Aim of the study

Here we examined associations between polypharmacy and TOI. We controlled this association for the potentially confounding effects of early-life predictors of cognitive and emotional impairment, such as childhood IQ and education [12], and late-life predictors of physical impairment, such as co-morbidities and BMI [6, 14]. We hypothesized that polypharmacy would be associated with increased impairment in a normal aging population sample independent of the effect of chronic disorders.

### Ethics approval

Approval was obtained from the NHS Grampian Local Research Ethics Committee with reference 03/0151(abc1936).

## Methods

### Population

The study population was drawn from members of the Aberdeen 1936 Birth Cohort (ABC1936), established in 1999 in Aberdeen, Scotland. This cohort had been fully described elsewhere [15]. In brief, mental ability of all participants was measured with the Moray House Test (MHT) at age 11 year as part of the Scottish Mental Survey of 1947 (SMS 47). 498 dementia free survivors of the SMS 47 aged around 64 year (between 63 and 68 year) were recruited into wave 1 of the ABC1936 cohort from 1999 to 2004. Complete neuropsychological, physical examination and the Short-Form-36 (SF36) health survey data were available for 341 participants and included in the current study.

### Participants’ health details

Participants were asked about their current medications with the questions “what medications are you on?” and “Could you please show me your latest prescription(s)?” We classified medications based on their therapeutic category using the British National Formulary (BNF). We also counted combined formulation based on their separate components, for example Co-codamol was counted as paracetamol and codeine phosphate.

We asked for details of current or previous diseases or health issues. These were recoded using the International Statistical Classification of Diseases and Related Health Problems 10th Revision (ICD-10) [16]. After recoding, chronic conditions were counted and weighted based on the Charlson’s co-morbidity index. Briefly, eighteen chronic conditions were identified. Myocardial infarction, congestive heart failure, peripheral vascular disease, cerebrovascular disease, dementia, chronic obstructive pulmonary disease, connective tissue disease, ulcer disease and diabetes were counted and weighted as 1. Hemiplegia, moderate or severe renal disease, diabetes with end organ damage, any tumor, leukaemia and lymphoma were weighted as 2. Moderate or severe liver disease were weighted as 3 and metastatic solid tumor and AIDS/HIV were weighted as 6. A score of co-morbidities was calculated based on this index [17]. Participants’ co-morbidity status was classified as 0 for not ill; 1 and 2 for mildly to moderately ill; and 3 or more for severely ill.

### Education achievement

Having no qualifications was coded 1; lower and higher leaving certificates were coded 2 and 3 respectively; Scottish vocational certificate coded 4; ordinary grades coded 5; Higher/Advanced level were coded 6; undergraduate and postgraduate degrees were coded 7 and 8 respectively and a professional or higher degree coded as 9 [15]. Educational achievement was used as a possible mediator of TOI in the model.

### Neuropsychological and cognitive tests

Cognitive ability of the participants at age 64 year was measured using four different tests including: Auditory Verbal Learning Test (AVLT), assessing verbal memory [18]; Digit Symbol Score (DS), a processing speed measure [19]; Block Design Score (BLK), as a spatial ability measurement [19] and Raven’s Progressive Matrices (RPM), a non-verbal reasoning measure of fluid intelligence [20]. Childhood mental ability at age 11 year (± 6 months) (MHT) was assessed by the Scottish Council for Research in Education in 1947.

### Physical and mental health examination

The SF36 health survey is a self-reported multi-dimensional health related quality of life questionnaire and consists of eight different domains [21]. We used the mean of Physical Functioning (PF), Role-Physical (RP), Bodily Pain (BP) and General Health (GH) domains as a self-assessed subjective measure of physical health of the participants. The mean of Vitality (VT), Social Functioning (SF), Role-Emotional (RE) and Mental Health (MH) domain scores were used as a measures of mental health. Walk time, the time in seconds it took participants to walk 6 m, normalised for height, and HADS depression and anxiety scores were analysed as indicators of physical and emotional function respectively.

### Triad of impairment

We used cognitive ability test results, and physical and mental health examinations to calculate our outcome of interest, TOI, using Principal Component Analysis (PCA). We reduced our variables of interest in two steps to obtain a single representative value for impairment in cognitive, physical and emotional function. We used the same method to calculate TOI as described previously [12] with some modifications. Briefly, the cognitive test scores, AVLT, DS, BLK and RPM, were used to calculate general cognitive factor (gf). HADS anxiety and depression score and the SF36 mental health domain scores were used to derive an emotional impairment factor (ef). A physical impairment factor was obtained using PCA on walk time and mean of the SF36 physical health domains (pf). In the second step, PCA was performed on gf, ef and pf to calculate the component TOI as an overall indicator of impaired cognition, physical and emotional function.

### Statistical analysis

Independent sample t-tests (continuous normally distributed variables) and Kolmogorov–Smirnov tests (non-normally distributed variables) were used to compare variables between genders. Due to a non-normal distribution TOI was log transformed before use as a dependant variable in the regression models. Statistical models were developed in two steps. First, a multiple regression model was used to examine associations between polypharmacy and TOI adjusting for co-morbidity score (IBM SPSS, 24). Age, gender, childhood IQ, education and BMI were included as co-variates of interest in the final model. Next, Structural Equation Models (SEM) were designed (IBM, AMOS, 24). We defined TOI in the SEM as a latent variable which is obtained from gf, ef, and pf. This approach enabled us to examine direct and indirect (mediating) contributions of medications and co-morbidity to latent TOI as well as to individual domains of latent TOI. We analysed number of medications used as a continuous variable in the SEM. This approach accounts for linear effects of number of medications (e.g. from 1 to 4) and a possible dose response relationship which would be lost using a binary classification system [22]. The goodness of fit was assessed according to the combined value of Comparative Fit Index (CFI) ≥ 0.95 and Root Mean Square Error of Approximation (RMSEA) ≤ 0.06 as an acceptable fit of the model to the data [23]. We examined modification indices in order to introduce new plausible paths to the model to improve model fit. The significance level for all relationships was α = 0.05.

## Results

### Gender differences

A summary of overall demographic, cognitive, physical and health data are shown in Table 1. Female participants scored higher on AVLT and DS tests whereas male participants performed better at BLK and walk time. In addition, female participants reported higher levels of anxiety (HADS anxiety) compared to male participants. TOI did not differ between genders.

**Table 1:** Characteristic of study population, comparison in predictors and outcome variables by gender

Characteristics	All N = 341	Female N = 171	Male N = 170
Age mean (SD)	64.8 (0.8)	64.9 (0.8)	64.7 (0.8)
*Number of medications n (%)*			
0	104 (30.5)	49 (28.6)	55 (32.3)
1–4	195 (57.2)	97 (56.7)	98 (57.6)
≥ 5 (polypharmacy)	42 (12.3)	25 (14.6)	17 (10.0)
Charlson’s co-morbidity index median (range)	1 (0–8)	1 (0–8)	1 (0–4)
*Charlson’s co-morbidity index n (%)*			
0 (not ill)	147 (43.1)	68 (39.8)	79 (46.5)
1–2 (mildly to moderately ill)	166 (48.7)	84 (49.1)	82 (48.2)
≥ 3 (severely ill)	28 (8.2)	19 (11.1)	9 (5.3)
Childhood IQ mean (SD)	44.2 (12.3)	45.2 (12.0)	43.2 (12.5)
Education mean (SD)	3.3 (2.3)	3.4 (2.3)	3.2 (2.3)
BMI mean (SD)	27.0 (4.2)	27.1 (4.8)	26.9 (3.5)
*Cognitive mean (SD)*			
Ravens progressive matrices (RPM)	36.8 (8)	36.6 (8.1)	36.9 (7.8)
Auditory verbal learning test (AVLT)	59.6 (12.8)	64.4 (11.6)**	54.8 (12.1)**
Digit symbol (DS)	44.9 (11.3)	47.1 (11.5)**	42.7 (10.6)**
Block design test (BLK)	25.2 (8.4)	23.4 (8.3)**	27.0 (8.2)**
*Physical mean (SD)*			
SF36 mean of physical health	75.1 (21.4)	75.9 (20.0)	74.4 (22.8)
6 m walk time test (s) (WTM)	3.0 (0.7)	3.3 (0.7)**	2.9 (0.6)**
*Emotional mean (SD)*			
SF36 mean of mental health	81.9 (17.1)	81.7 (16.8)	82.0 (17.5)
HADS anxiety	5.8 (3.1)	6.4 (3.2)**	5.2 (2.8)**
HADS depression	2.9 (2.3)	3.0 (2.4)	2.8 (2.2)
Triad of impairment mean (SD)	69.1 (8.3)	69.8 (8.4)	68.4 (8.1)

*p ≤ 0.05, **p ≤ 0.001

### Extraction of derived physical, emotional and cognitive factors and TOI

Results of the PCA, percentage of total variance explained by the new extracted component and component loading for the variables are given in Table S1 Supplementary data. The first un-rotated principal component (gf) described 55% of the shared variance between cognitive tests. The component “ef” explained 66% and the component “pf” accounted for 68% of the total variance. The component loadings for gf, ef and pf were comparable in the new calculated component, TOI (Table S1, Supplementary data).

### Association between polypharmacy and TOI

Table 2 shows the relationship between polypharmacy and TOI adjusting for co-morbidity score and other confounding variables. Polypharmacy was associated with increased TOI even after adjusting for co-morbidity score and other confounders (β = 3.6, *p* = 0.003). As expected increased co-morbidity score was associated with increased TOI and this effect is stronger for the severely ill participants (β = 5.3 and β = 2.5 for severely and mildly to moderately ill respectively, not ill set to 0 as control, *p* < 0.01).

**Table 2:** Regression models examine the effect of polypharmacy on TOI score (TOI range: 48.8–96.4, increased in the score means increased impairment, n = 341)

Characteristics	Unadjusted model		Adjusted model^a^		Adjusted model^b^	
	β (95% CI)	*p* value	β (95% CI)	*p* value	β (95% CI)	*p* value
Intercept	68.3	< 0.001	66.3	< 0.001	87.3	0.003
0–4 medicines	0		0		0	
≥ 5 medicines (polypharmacy)	5.9 (3.3, 8.6)	< 0.001	4.3 (1.5, 7.0)	0.002	3.6 (1.2, 6.0)	0.003
*Co-morbidity score*						
0	–		0		0	
1–2 (mildly to moderately ill)	–		3.7 (1.9, 5.4)	< 0.001	2.5 (0.9, 4.1)	0.002
≥ 3 (severely ill)	–		5.7 (2.3, 9.1)	0.001	5.3 (2.3, 8.3)	0.001
Age	–		–		− 0.2 (− 1.1, 0.6)	0.565
*Gender*						
Male	–		–		0	
Female	–		–		1.5 (− 0.1, 2.9)	0.046
Childhood IQ	–		–		− 0.2 (− 0.3, − 0.1)	< 0.001
Education	–		–		− 0.8 (− 1.2, − 0.4)	< 0.001
BMI	–		–		0.2 (0.1, 0.4)	0.007
R^2^	0.056		0.113		0.353	
Adjusted R^2^	0.053		0.105		0.337	

^a^Adjusted for co-morbidity score

^b^Adjusted for co-morbidity score, age, gender, childhood IQ, education and BMI

Lower childhood IQ, lower educational qualification and higher BMI were associated with increased impairment in cognitive, physical and emotional capability (Table 2).

### Structural equation modelling

**Fig. 1:**
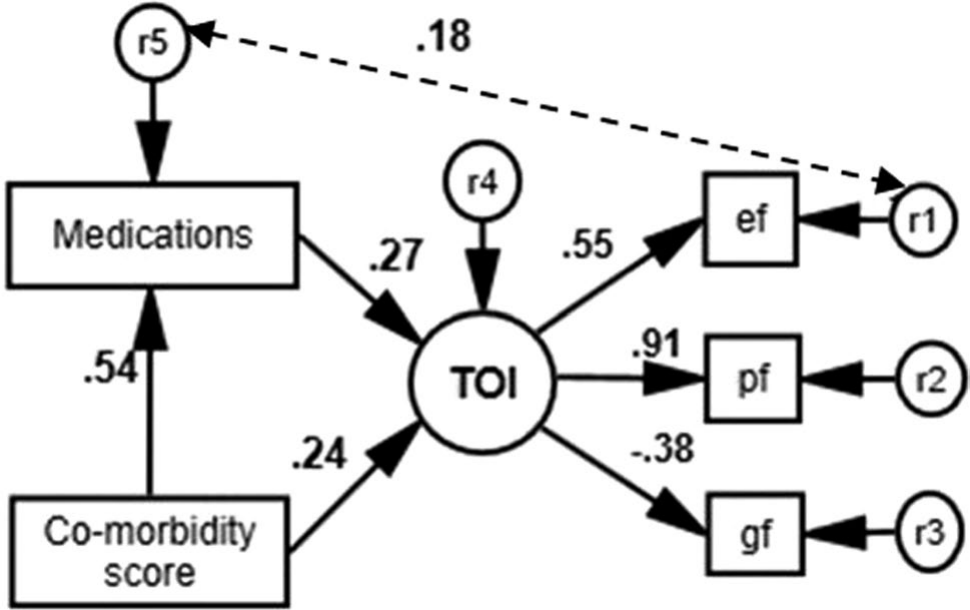
Path diagram of hypothesised model (n = 341). Solid lines represent hypothesised path and dash line represent path added to the model based on the modification indices. *ef* emotional impairment factor, *pf* physical impairment factor, *gf* cognitive ability factor. All the standardised regression weights represents in the diagram are statistically significant (*p* < 0.001). r1 to r5 are residual (error) terms within the model

Figure 1 shows the path diagram and the results of the SEM. Pf had the highest loading factor of 0.91 in the new calculated component, TOI. The model had good fit indices as CFI = 0.993 and RMSEA = 0.048.

As expected, co-morbidity contributed significantly to mean number of medications (standardised β = 0.54, *p* < 0.001). Mean number of medications contributed directly to latent TOI (standardised β = 0.27, *p* < 0.001). Co-morbidity had a direct effect on TOI (standardised β = 0.24, *p* < 0.001). The indirect effect of co-morbidity on TOI was mediated through increased number of medications (standardised β = 0.15). The standardised total contribution of increased co-morbidity to TOI was β = 0.39. After an initial analysis, modification indices indicated that a new correlation between r1 and r5 would improve model fit (Fig. 1). This correlation (correlation coefficient = 0.18, *p* = 0.004) suggests that an individual’s emotional impairment and mean number of medications error term could be affected by the same unmeasured common factor.

We then excluded participants that reported any affective disorders (e.g. depression and anxiety) and/or were taking any related medications such as selective serotonin reuptake inhibitors, tricyclic antidepressants and benzodiazepines to eliminate the effects of these disorders on the relationships described in our model. We then replicated the analysis in the remaining 303 participants and found the same results as for the whole sample. The results of this model are available in the Supplementary data (Figure 2S, Supplementary data).

## Discussion

### Interpretation of the results

Here we showed that polypharmacy was associated with increased impairment in cognitive, physical and emotional capability in a dementia-free aging sample of the general population. Increased co-morbidity and a high BMI were also related to increased impairment in this population. As expected, higher childhood IQ and educational qualification were negatively associated with TOI.

The most striking finding in this study was the association of polypharmacy and increased impairment in late life, independent of chronic diseases and other possible risk and protective factors. This finding demonstrates that considering number of medications taken, in addition to morbidity measurements, would improve our understanding of functional health status in older adults.

Our findings are consistent with the longitudinal British birth cohort study that found a significant relationship between polypharmacy and cognitive and physical impairment. In this study stronger negative associations were seen in participants with longstanding polypharmacy, suggesting a cumulative, dose-dependent association, where dose is the number of prescribed medications [6]. It is plausible that number of medications plays a causal role in increasing TOI in older adults. Recently the role of polypharmacy in increasing risk of frailty and impairment has been of much research interest. Recent studies have found that polypharmacy is associated with higher prevalence of frailty due to medicine interactions, malnutrition and more severe adverse effects in older people [3, 4, 24]. Further research should establish whether a higher TOI score is an early manifestation of frailty development and if polypharmacy is associated with an increased prevalence of frailty syndrome in this population. It is notable that polypharmacy could be both necessary and rational in some older adults with multimorbidity and frailty. It is therefore important to distinguish and consider appropriate from inappropriate polypharmacy when discussing its outcomes [2].

Although the co-morbidity had a strong positive effect on number of medications (β = 0.54, *p* < 0.001), we found that number of medications and emotional impairment could possibly be affected by the same unmeasured factor. This interesting finding could suggest that poorer emotional capability is associated with increased use of general and mental health services, and thus increased medication use [25]. Increased risk of mental health disorders, such as depression and anxiety, might also lead to higher medication use. However, our model finds that this relationship is significant in participants that did not report any neurotic disorder or related medicine use (Figure 2S, Supplementary data). A possible explanation could be that undiagnosed depression and anxiety increases self-medication in older people [e.g. taking of over the counter (OTC) medications] and increases the prevalence of polypharmacy [26].

### Strengths and weaknesses

Although physical, emotional and cognitive impairment often co-exist in the elderly and recent studies showed that they may have shared origins [9, 10, 27], their co-occurrence is rarely investigated [11, 12]. TOI used in this study enabled us to study the co-occurrence of impairment in multiple domains, including physical deficits. The key strengths of our study include using a wide range of predictors of cognitive and physical impairment from early-life to late mid-life. To test possible causal relationships between these inter-related factors we used robust statistical methods and modelling. Improving our understanding about how these predictors are associated with TOI allows us to identify targets that might reduce impairment in older adults.

Weaknesses are the relatively small sample size and the use of self-reported medications and description of diseases which could introduce recall bias. However, the participants were relatively young and dementia-free when data were collected which would reduce the likelihood of misclassification or under/over-estimation of these results. We also only measured TOI, medications and morbidities at a single time point. Longitudinal research is needed to understand the casual relationships and to make prediction of future cognitive, emotional and physical function in this population.

## Conclusion

Polypharmacy is associated with increased impairment in cognitive, physical and emotional function in older adults. This relationship is independent of the co-morbidity burden and other confounding factors. Longitudinal studies are required to better understand the underlying mechanisms of the observed relationships in this study. Our findings suggest that reducing inappropriate polypharmacy is a promising strategy to reduce impairment in different domains in older adults and associated healthcare costs.

## Electronic supplementary material

Below is the link to the electronic supplementary material.

Supplementary material 1 (DOCX 102 kb)
