# Supplementary material for: The association between polypharmacy and late life deficits in cognitive, physical and emotional capability: a cohort study
Source: Int J Clin Pharm. 2018 Nov 29;41(1):251–7. doi: 10.1007/s11096-018-0761-2 (PMC6394523; doi:10.1007/s11096-018-0761-2)

# Supplementary data

Table S1. Principal Component Analysis to calculate the single measure of impairment (TOI) using cognitive, physical and emotional capability, percentage of shared variance and components’ load

| Factor | % of variance | Components | Load |
| --- | --- | --- | --- |
| general cognitive factor (gf) | 54.8 | RPM | 0.83 |
|  |  | DS | 0.78 |
|  |  | BLK | 0.76 |
|  |  | AVLT | 0.54 |
|  |  |  |  |
| emotional impairment factor (ef) | 65.5 | SF 36 MH | -0.83 |
|  |  | HADS-A | 0.77 |
|  |  | HADS-D | 0.82 |
|  |  |  |  |
| physical impairment factor (pf) | 67.8 | SF 36 PH | -0.82 |
|  |  | WTM | 0.82 |
|  |  |  |  |
| triad of impairment (TOI) | 59.4 | gf | -0.65 |
|  |  | ef | 0.80 |
|  |  | pf | 0.84 |
| RPM, Raven’s Standardized Progressive Matrices; DS, Digit Symbol Test; BLK, Block Design Test; AVLT, Rey’s Auditory Verbal Learning Test; SF 36 MH, SF36 health survey mean of Mental Health domains; HADS-A, HDAS Anxiety score; HADS-D, HADS Depression scorer; SF 36 PH, SF36 health survey mean of Physical Health domains; WTM, Walk time in second to take 6 meters, normalized for height | | | |

**Figure**

Supplementary Figure 1. Path diagram of hypothesised model in participants with no CNS disorders, and /or no CNS related medications (n=303). Solid lines represent hypothesised path and dash line represent path added to the model based on the modification indices. ef, emotional impairment factor, pf, physical impairment factor; gf, cognitive ability factor. All the standardised regression weights represents in the diagram are statistically significant (p < 0.001). r1 to r5 are residual (error) terms within the model. (The standardised total effect of co-morbidity score on TOI is (0.53×0.24) +0.29=0.42 p<0.001)


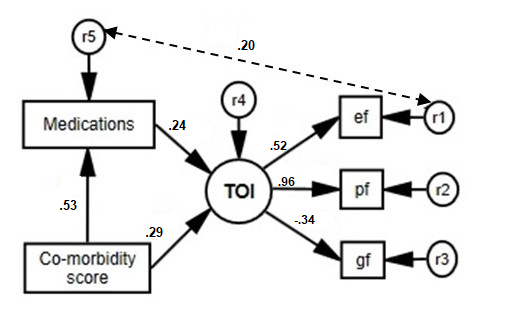

Supplement: Supplementary file 1 — Supplementary material 1 (DOCX 102 kb) [file 11096_2018_761_MOESM1_ESM.docx]
